# Scaling up specialist training in developing countries: lessons learned from the first 12 years of regional postgraduate training in Fiji – a case study

**DOI:** 10.1186/1478-4491-10-48

**Published:** 2012-12-27

**Authors:** Kimberly Oman, Elizabeth Rodgers, Kim Usher, Robert Moulds

**Affiliations:** 1 Consultant Infectious Diseases Physician, The Townsville Hospital, 100 Angus Smith Drive, Townsville, QLD 4814, Australia; 2 Head of the Department of Medical Sciences, College of Medicine, Nursing and Health Sciences, Fiji School of Medicine, Fiji National University, Private mail bag, Suva, Fiji; 3 Discipline of Nursing, Midwifery and Nutrition, James Cook University, Cairns Campus, PO Box 6811, Cairns, QLD 4870, Australia; 4 Medical Advisor to Therapeutic Guidelines, Therapeutic Guidelines Limited, 473 Victoria St, West Melbourne, VIC 3003, Australia

**Keywords:** Education, Medical, Postgraduate, Developing countries, Pacific Islands, Human resources for health, Professional satisfaction, Case study, Qualitative research, Medical migration, Mixed methods research

## Abstract

**Background:**

In 1997, regional specialist training was established in Fiji, consisting of one-year Postgraduate Diplomas followed by three-year master’s degree programs in anesthesia, internal medicine, obstetrics/gynecology, pediatrics and surgery. The evolution of these programs during the first 12 years is presented.

**Case description:**

A case study utilizing mixed methods was carried out, including a prospective collection of enrolment and employment data, supplemented by semi-structured interviews. Between 1997 and 2009, 207 doctors (113 from Fiji and 94 from 13 other countries or territories in the Pacific) trained to at least the Postgraduate Diploma level. For Fiji graduates, 29.2% migrated permanently to developed countries, compared to only 8.5% for regional graduates (*P* <0.001). Early years of the program were characterized by large intakes and enthusiasm, but also uncertainty. Many resignations took place following a *coup d*’*etat* in 2000. By 2005, interviews suggested a dynamic of political instability initially leading to resignations, leading to even heavier workloads, compounded by academic studies that seemed unlikely to lead to career benefit. This was associated with loss of hope and downward spirals of further resignations. After 2006, however, Master’s graduates generally returned from overseas placements, had variable success in career progression, and were able to engage in limited private practice. Enrolments and retention stabilized and increased.

**Discussion and evaluation:**

Over time, all specialties have had years when the viability and future of the programs were in question, but all have recovered to varying degrees, and the programs continue to evolve and strengthen. Prospective clarification of expected career outcomes for graduates, establishment of career pathways for diploma-only graduates, and balancing desires for academic excellence with workloads that trainees were able to bear may have lessened ongoing losses of trainees and graduates.

**Conclusions:**

Despite early losses of trainees, the establishment of regional postgraduate training in Fiji is having an increasingly positive impact on the specialist workforce in the Pacific. With forethought, many of the difficulties we encountered may have been avoidable. Our experiences may help others who are establishing or expanding postgraduate training in developing countries to optimize the benefit of postgraduate training on their national and regional workforces.

## Introduction

The World Health Organization has estimated that there is a global shortage of over 2.5 million health care workers. These shortages are most acute in developing countries, where an important approach to increasing the numbers of health workers is the “scaling up” of health professional education and training [1,2], including the establishment of in-country and regional specialist training [3].

With support from the Australian government, regional postgraduate specialist training was established in the late 1990s in Fiji, a small developing Pacific Island nation. This was done in order to address the failure of most overseas-trained Pacific Island specialists to return to or remain in the Pacific, along with an ongoing dependence on expatriates for specialist services in the region [4-7]. The early years after the establishment of these programs, however, coincided with an era of increasing openness of developed countries, including nearby Australia and New Zealand, to the migration of doctors from developing countries. In Australia, from the mid-1990s to the mid-2000s, there was a progressive liberalization of temporary entrant requirements for overseas trained doctors that led to increasing numbers who were recruited through “area of need” visas to fill workforce requirements in outer metropolitan, rural and remote locations, as well through occupational trainee visas to work in public hospitals [8] (p. 24–25)]. Several new nationally standardized registration pathways were introduced in 2008 to further facilitate entry to medical practice [9]. This was compounded by a disruptive *coup d*’*etat* in Fiji in 2000. Within a few years, concerns were being raised about unexpected levels of resignations of specialist trainees and former trainees from the public sectors in Fiji, often to migrate. Because of these concerns, a longitudinal study was instituted in 2004 which incorporated ongoing tracking of trainee and graduate career outcomes along with individual interviews which focused on exploring the reasons for professional satisfaction, dissatisfaction and career decision making.

We describe here the evolution of postgraduate specialist training in the Pacific over the past 12 years, and identify a number of lessons learned, especially from the difficulties that arose in the early years of the course. These lessons may be of benefit to aid agencies, institutions and educators who are considering the support and establishment of national or regional postgraduate training programs in other developing countries.

### Case description

#### Data collection and evaluation

Data collection and ongoing evaluation of the programs included quantitative data that were collected over a number of years (updated in September 2010) on doctors who had undertaken postgraduate studies at the Fiji School of Medicine (FSMed) to at least the postgraduate diploma level between 1998 (1997 for anesthesia) and 2009. Data included country of origin, gender, race, year of postgraduate training, highest qualification attained and current working situation. Most data came from senior specialists, supplemented by enrolment and graduation records. Data were entered and analyzed using Epi-info [10], with chi-square testing used for comparisons.

The study presented here is part of a larger exploratory mixed-methods case study [11-13], for which the methodology is described in detail elsewhere [14-16]. Briefly, interviews were carried out between 2004 and 2006 with 36 out of 66 Fiji doctors who had completed at least one year of training by December 2004, along with a number of senior specialists. The semi-structured interviews focused on professional satisfaction, dissatisfaction and career decisions. Interviews were analyzed using a constant comparative method that identified and tested emerging themes [17,18]. While this study does not present new findings based on the qualitative interviews that have been explored in previous publications [14-16,19], it presents these findings in an accessible narrative fashion that clarifies and gives context to several additional years of both longitudinal data and further descriptions of the evolution of the programs.

Evaluation of the programs was carried out on an ongoing basis with feedback of both qualitative and quantitative findings presented to key clinical and Ministry of Health stakeholders at Fiji Medical Association annual conferences in 2005, 2006 and 2007. Publications based on the case study [14-16,19] as well as the unpublished PhD thesis [13] were also made available to key stakeholders. This study received ethical approval from the Fiji National Research Ethics Review Committee and from James Cook University (Townsville, Australia).

#### Case description - postgraduate trainees from Fiji

Postgraduate training at the Fiji School of Medicine (FSMed) consists of a one-year postgraduate diploma followed by three more years of a master’s program in medicine training [20-23], and can be undertaken in anesthesia, internal medicine, obstetrics and gynecology, pediatrics, and surgery. Applicants are required to have an MBBS or equivalent degree, which for most Fiji and regional trainees is awarded by the Fiji School of Medicine. Applicants are further required to complete a one-year internship and two years of medical service roles in the Pacific, of which one year is required to be in the specialty they are applying for (this is sometimes waived for applicants from countries without the prerequisite specialist services). Trainees are selected by the health services of their home countries, and the local medical councils for each country are responsible for developing policy in regards to the specialist status of graduates.

The programs were established through an $AUD 5.5 million dollar (approximately $US 5.5 million dollars) five-year grant funded by AusAID [4], and contracted through the Royal Australasian College of Surgeons. The programs were approved by the Ministry of Health and accredited by the University of the South Pacific. This supported one external specialist advisor from Australia or New Zealand in each of the five specialties. These advisors worked alongside a local specialist counterpart for three years in Fiji (with subsequent follow-up visits) to develop local curricula. The specialist colleges for Australia and New Zealand provided varying levels of support, but no formal qualifications, and there were no local specialist societies with the authority to award formal qualifications. The first year was designed as basic stand-alone specialty training with weighting towards local patterns of disease, and included an exit exam and the awarding of a Diploma qualification. The following three years of Master’s training could be entered with a “B” grade or above in the Diploma course, and included a curriculum that was strongly influenced by Australasian curricula but again weighted towards the local spectrum of disease. Additional common modules in public health and basic sciences were also developed. An exam was taken in the second Master’s year, and a required project completed in the third year. During this third year, many trainees undertook overseas attachments in Australia or New Zealand that were either employed training or observer roles. Ongoing financial support of the programs has been principally through course fees that are funded mainly by Ministries of Health and aid agency scholarships.

Between 1997 and 2009, 207 doctors trained to at least the Postgraduate Diploma level at the Fiji School of Medicine (FSMed), including 113 Fiji doctors (39.8% female) and 94 doctors from 13 other countries or territories in the region (31.9% female). Of these doctors, 50 (25 each from Fiji and regional countries) had been awarded Master’s in Medicine degrees, and by mid-2010, 48 others were either undertaking Master’s training (41) or were believed to be awaiting a post-Diploma training position (7) (see Table 1).

**Table 1:** **Highest qualification**^**a**^**of enrollees in postgraduate specialist training at the Fiji School of Medicine, 1997 to 2009**

**Country or territory of residence**	**Population[**[1,][48]**]**	**Diploma**-**only graduates**^**b**^	**Master’s students**^**c**^	**Master’s graduates**	**Total enrollees**
American Samoa	65,000 [49]	3	0	2	5
Cook Islands	20,000	4	0	1	5
East Timor	1,134,000	1	1	0	2
Federated States of Micronesia	111,000	6	1	3	10
Kiribati	98,000	9	0	1	10
Marshall Islands	62,000	2	0	0	2
Niue	<1,0000	0	1	0	1
Palau	20,000	2	0	2	4
Samoa	179,000	9	3	0	12
Solomon Islands	523,000	3	4	7	14
Tonga	104,000	3	3	6	12
Tuvalu	10,000	5	1	0	6
Vanuatu	240,000	4	4	3	11
Total enrollment – other than Fiji		51	18	25	94
Fiji	849,000	58	30	25	113
Total Enrollment		109	48	50	207

^**a**^as of September 2010.

^**b**^enrollees who left training with a Postgraduate Diploma as their highest qualification. Several of these obtained Postgraduate Diplomas at institutions other than the Fiji School of Medicine and subsequently enrolled in Master’s training but did not complete a Master’s qualification.

^**c**^including Master’s students who have deferred studies (1) and Postgraduate Diploma graduates awaiting Master’s enrollment (6).

#### Postgraduate trainees from Fiji

The earliest years of formal postgraduate training in Fiji were characterized by large yearly intakes (14 to 18) to accommodate doctors who were working in specialty departments but had never had the opportunity to undertake formal specialist training (see Table 2).

**Table 2:** Number of trainees who were awarded a specialist Postgraduate Diploma or Master’s in Medicine at the Fiji School of Medicine by year

**Year**	**Postgraduate Diploma awarded**			**Master’s in Medicine awarded**		
	**Fiji**	**Other Pacific Islands**	**Total**	**Fiji**	**Other Pacific Islands**	**Total**
1997	4	2	6			
1998	14	9	23			
1999	9	7	16			
2000	5	4	9			
2001	5	10	15	4	2	6
2002	6	5	11	6	3	9
2003	10	6	16	4	3	7
2004	4	7	11	2	1	3
2005	7	9	16	3	4	7
2006	5	6	11	2	3	5
2007	11	6	17	1	1	2
2008	9	8	17	0	4	4
2009	15	11	26	3	4	7
Total	104	90	194	25	25	50
(Diploma awarded elsewhere^a^)	(9)	(4)	(13)			

^**a**^nine Fiji and four regional Master’s in Medicine students who received their Postgraduate Diplomas at institutions other than the Fiji School of Medicine subsequently enrolled in Master in Medicine training.

In May 2000, a disruptive *coup d*’*etat* took place in Fiji that had considerable impact on the entire health system. Many Diploma graduates dropped out of the specialist Master’s training, and most of these left the govern-ment health service, usually to undertake employment in developed countries, which at the time were actively recruiting overseas trained doctors. Losses of trainees continued over the next few years, and raised concerns not only about the viability of the postgraduate programs, but about the wisdom of running a training program that seemed to mainly benefit the country that had funded the establishment of the training. Data from early 2005 revealed that more Diploma graduates were residing either perma-nently or temporarily in developed countries (25) than were working in Fiji (23) in the public sectors (the Ministry of Health, Fiji School of Medicine or UN agencies) [24].

Interviews with senior specialists, specialist trainees and local graduates were carried out between 2004 and 2006, are summarized here, and are analyzed in detail elsewhere [14-16]. These interviews explored, among other things, the reasons for the low rates of retention of Fiji doctors in specialist training and in the public sectors, and identified forces at work that were arguably modifiable and within the control of the health system.

Interview participants described the early years after the establishment of the courses in 1998 as being times of excitement and hope, with initial large cohorts, but also as times of uncertainty. By the time of the 2000 *coup d*’*etat*, many trainees were struggling to balance unexpectedly high academic demands on top of already heavy clinical workloads. Some doctors migrated soon after the *coup d*’*etat*, usually due to concerns about the impact of political instability on their families, and this left higher workloads for those who remained, many of whom struggled to cope.

The first Master’s qualifications were awarded in 2001. Over the next few years their career outcomes were closely observed by their peers, and this led to discouragement and discontent. By 2004 and 2005, many interview participants described being aware of instances of unfairness in the promotion process, including favoring seniority over qualifications or performance, seemingly inexplicable delays in “deserved” promotions, as well as perceptions of broken promises in regards to the apparent lack of impact of Master’s and Postgraduate Diploma qualifications on career progression, which did not seem to lead to any benefits career-wise. A few early Master’s graduates had resigned from the public system to either migrate or to enter local private practice, and there were concerns that the Master’s graduates and students who were at the time training overseas might also decide not to return.

Postgraduate Diploma graduates who were considering whether to continue training often described struggling to cope with the demands of a heavy clinical workload on top of the demands of starting and raising young families on low salaries. This was discouraging in itself, but was compounded by some degree of loss of hope that adding academic studies to an already overburdened working life would lead to future benefit, given the disappointing career outcomes observed for the earlier Master’s graduates. Interestingly, there was no significant difference in the rates of resignations between men and women at that time (55.3% vs 44.7% respectively, *P* = 0.39), though men were more likely to cite financial pressures and women were more likely to cite a need for more time to raise their children.

Overall, by 2005 the interviews suggested a dynamic of political instability leading to resignations leading to even heavier workloads, compounded by academic studies that seemed unlikely to lead to career benefit. This led to loss of hope and downward spirals of further resignations, heavier workloads and further discouragement. On a personal level, out of 25 public sector doctors interviewed, 15 (60%) expressed unhappiness with their own career progression, and 10 (40%) described times when they had given serious consideration to resigning. Detailed analysis in late 2006, by which time 66 Fiji enrollees had received at least a Diploma, demonstrated that a majority (63.6%) had left training with a Postgraduate Diploma as their highest qualification (42 of 66). This was of concern because only 22.7% (15) of these Diploma graduates had remained in the public sectors (14) or were temporarily training overseas (1), while 28.8% (19) were believed to have permanently migrated. By comparison, 18 out of 21 (85.7%) Master’s graduates were still either in the public sector or temporarily overseas. It was a further point of concern that resignations and migration continued among Postgraduate Diploma graduates who had started their training after the 2000 *coup d*’*etat*, suggesting that despite the stabilization of the political situation, the factors driving resignations and migration were ongoing.

On a positive note, however, an overwhelming majority of these doctors, even those who had resigned, spoke of the satisfaction and fulfillment that they were able to gain through public sector work in spite of difficult working conditions, as well as an overall preference for public sector work.

By 2006, a number of positive developments were taking place, and these were reflected in the overall tone of the interviews. A number of Master’s students and graduates had returned to Fiji after their overseas attachments and settled into public sector roles. While unhappiness about the promotion process continued, a number of Master’s graduates, though not all, had experienced career advancement. Some Master’s graduates in the public sector had become eligible to undertake limited private practice, which was seen as bringing welcome financial relief.

Postgraduate enrollments increased significantly from 2007 to 2009. Enrollees from 2005 onward (47) were much more likely to be retained as Master’s students (61.7% compared to 39.4% in earlier years), although those who left training with a Postgraduate Diploma as their highest qualification still had low retention in the public sector (33.3% compared to 25.0% in earlier years). Factors that may have contributed to higher numbers of enrollees and improved retention may have included a more realistic understanding by prospective trainees of what training would involve, fewer training opportunities in Australia related to larger numbers of local graduates [25], and renewed hope that a Master’s qualification would be likely to benefit one’s career.

By mid-2010, 25 Fiji trainees had been awarded a specialist Master’s, and of these, only 4 are believed to have been lost permanently from the public sectors. While three others are currently undertaking training overseas, the trend in later years has been for Master graduates to return to Fiji after overseas training. The progress across the specialties has been uneven, however, and in some cases relates to a number of years of low enrollments in the earlier years of the course. In particular, some specialties remain short-staffed, and struggle with high teaching and clinical loads while waiting for existing trainees to graduate. Nevertheless, the programs have become well established, and while trainees and graduates are still being lost from the public system, the rate of this is less than in previous years. Overall, there is strong and ongoing support for these postgraduate programs from the Ministry of Health, which continues to fund individual trainees, from senior hospital specialists who are pleased with the quality of the graduates and their positive impact on the local specialist workforce, and from the Master’s graduates themselves who have expressed overall satisfaction with their training.

#### Case description - postgraduate trainees from other Pacific Islands

The experiences of the 94 regional enrollees have differed somewhat from those from Fiji. Enrollments have remained steadier over the years (5 to 11 per year) and have not demonstrated the initial large cohorts, low numbers in middle years followed by a catch-up period that has characterized postgraduate training for Fiji doctors. While a similar percentage of regional doctors left training with a Postgraduate Diploma as their highest qualification (54.3% as compared with 51.3% for Fiji), their retention in the public sector (including doctors who were temporarily residing in developed countries) was considerably higher (84.3%, or 43 of 51) than in Fiji (27.6%, or 16 of 58) (*P* <0.001). On the other hand, in-country retention of Master’s graduates was similar in regional countries (68.0% or 17 of 25) compared to Fiji (84.0% or 21 of 25) (*P* = 0.19). Interestingly, 6 of 25 (24.0%) regional Master’s graduates were working in the public sectors of Pacific Island nations outside of their home countries. This could reflect a situation where in smaller countries, there is a more defined role for Diploma graduates, while Master’s graduates may, in some cases, be either more easily assimilated into the healthcare systems or may be more “marketable” outside of their home countries. Interestingly, only 8.5%, or 8 of 94 regional graduates are believed to be permanently living in developed countries compared to 29.2% (33 out of 113) for Fiji (*P* <0.001), which is a particularly promising outcome (see Table 3).

**Table 3:** Work situation in 2010 for Fiji School of Medicine specialist graduates (1997 to 2009 intake cohorts)

**Training status**	**Total**	**Retained in home country public sectors**		**Retained in the Pacific outside of home country public sectors**		**Permanent migration to a developed country**
		**Public sectors**^**a**^**in home country**	**Developed country(temporary)**	**Private practice in home country**	**Pacific outside of home country**	**Developed country(long term)**
All Fiji graduates	113	63 (55.8%)	4 (3.5%)	9 (8.0%)	4 (3.5%)	33 (29.2%)
Fiji Master’s graduates	25	18 (72.0%)	3 (12.0%)	1 (4.0%)	0 (0.0%)	3 (12.0%)
Fiji Diploma-only graduates^b^	58	16 (27.6%)	0 (0.0%)	8 (13.8%)	4 (6.9%)	30 (51.7%)
Fiji current Master’s students^c^	30	29 (96.7%)	1 (3.3%)	0	0	0
All regional graduates	94	58 (61.7%)	5 (5.3%)	1 (1.1%)	22 (23.4%)	8 (8.5%)
Regional Master’s graduates	25	14 (56.0%)	3 (12.0%)	0 (0.0%)	6 (24.0%)	2 (8.0%)
Regional Diploma-only graduates^b^	51	41 (80.4%)	2 (3.9%)	1 (2.0%)	1 (2.0%)	6 (11.7%)
Regional current Master’s students^d^	18	3 (16.7%)^d^	0	0	15 (83.3%) – training in Fiji	0

^a^For Fiji, this includes the Ministry of Health, the Fiji School of Medicine or a regional / international public health agency.

^b^Excluding current Master students, deferred students or students awaiting Master’s enrollment following the awarding of a Postgraduate Diploma.

^c^Including four Postgraduate Diploma graduates awaiting Master’s enrollment.

^d^Including two Postgraduate Diploma graduates awaiting Master’s enrollment, and one deferred Master enrollee.

### Discussion and evaluation

In the existing literature, “scaling up” of medical training has been proposed as a means of addressing the shortages of health workers in developed countries [1,26], and it has been proposed that doctors who train in-country or regionally are more likely to be retained than those who train in developed countries [3]. This study provides support for the potential of in-country and, in particular, regional training programs to deliver positive impacts for specialist workforces in developing countries. In the Pacific, after 12 years, 50 specialists have been trained to the Master’s level, and their retention in the Pacific has been excellent. Another 157 doctors have attained Diplomas, of whom almost one-third are current Master’s students. Retention of Diploma graduates outside of Fiji has also been excellent, but in Fiji itself, losses of Diploma graduates from the public sector have been disappointing and ongoing, despite the prospect of increasing barriers to training in Australia and New Zealand in recent years related to the increasing numbers of local medical graduates [25]. Overall, retention in Master’s training in Fiji has improved over the years, which is a positive development, and is likely to end up having a positive overall impact on retention in the public sectors. Already, most hospital specialists are local graduates, with several graduates being employed in senior roles at the Fiji School of Medicine. Pacific Island Ministries of Health continue to enroll specialist trainees, and the growing number of enrollees and graduates indicates strong and ongoing strong support for these programs.

This study has a number of strengths and weaknesses. Data on graduates have been gathered and updated regularly since 2004, and are reasonably accurate and complete, especially for Fiji doctors, though less so in particular for earlier regional graduates. Whereabouts and, in particular, the permanency of migration can also be difficult to assess with absolute certainty, and depends on impressions of colleagues. There are also no solid data on the number of Diploma graduates who did not continue because of eligibility or performance issues, and this is related to the availability of the option of repeating the Diploma and other years of the programs for ineligible trainees, as well as uncertainty in regards to why these options may not have been undertaken. Limitations of the qualitative aspects of this study are discussed elsewhere [14-16].

A particular strength of this study is that it provides rich qualitative data on career decisions in a multi-speciality postgraduate training program, as well as actual career outcomes over a 12-year period. Other studies on postgraduate retention from Ghana [27] and China [28] have focused on reasons for remaining or resigning, respectively, but do not document career outcomes over time. Previous descriptive studies on postgraduate training in developing countries have generally not focused on retention issues [29-34]. Other studies from developing countries have focused on doctor motivation [35-37], dissatisfaction [37-41], coping mechanisms [42-44] and reasons for migrating or considering migration [45-47], but have not explored these issues in the context of postgraduate training.

Our experiences point to a number of “lessons learned” for aid agencies and national health ministries, as well as educational institutions, professional societies and educators who collaborate to establish, develop and deliver these programs. If others are able to anticipate these issues and proactively prepare their health systems for the graduation of the first cohorts of trainees, they may be able to avoid some of the problems we encountered. These issues, which are complex and often have no “right” or “wrong” answers, include unintentional undermining of programs by donor countries, differing expectations about the impact of specialist qualifications on career advancement leading to disappointment and disillusionment, the dilemma of developing rigorous courses that nonetheless have a workload that trainees are able to bear, as well as the lack of clear and desirable career options within the public sectors for doctors who are unable to complete training.

From the standpoint of aid agencies in developed countries, there is strong evidence that devoting resources to “scaling up” specialist numbers through the establishment of in-country and, in particular, regional training can lead to potentially long lasting impacts on workforce sustainability. On the other hand, one of the greatest threats to these programs, especially in Fiji, has been the openness of Australia and New Zealand to employ graduates of these programs, whose level of training in a program designed with strong input from Australian or New Zealand specialists, made them particularly attractive. While this may prove to be less of a problem in the future with increases in the numbers of Australian medical graduates, aid agencies in donor countries should be aware of any national policies that could undermine the training programs that they are establishing. Increasing the number of medical graduates in developed countries to the point where local needs are met is likely to have a positive impact on the retention of locally trained basic medical and specialist graduates in developing countries,

Career pathways and advancement are areas that require particular attention. The early years of new courses are likely to be full of uncertainties, and the quality of the graduates will initially be unclear. There is a very real risk that until they have “proven themselves”, graduates may be undervalued compared to overseas-trained specialists or expatriate recruits. Early on, particular scrutiny is likely to be paid to the career outcomes of early graduates, and perceptions of unfairness in the promotions process could lead to discouragement, resignations, and undermining of new training programs that are being established. The earliest years of new courses, and indeed the planning stages, are times to openly discuss how local specialist qualifications should be counted in promotions processes. Agreement should also be sought from national registering authorities about the impact of local specialist training on attaining nationally recognized specialist status. Such discussions were held in Fiji, but the failure to document the outcomes of these discussions in writing led to disappointment and dissatisfaction among local graduates related to perceptions of unmet expectations. Decisions about the impact of training on career progression are complicated by the broad range of seniority among early enrollees, from relatively junior to highly experienced in specialist fields but without formal training. The dilemma of highly experienced doctors who cannot be spared to undertake specialist training or who do not see its value should also be considered. In early years, beliefs will develop about the impact of training on career advancement, and it is important to avoid making or implying promises that can not be kept. The years of the course surrounding the first graduations is a time when transparency in the promotions process, attention to justice and fairness, and avoidance of delays in filling established posts could be expected to be of particular value. It may be of benefit to tie the attainment of a Master’s or Postgraduate Diploma qualification to automatic career advancement. The extra funding required for this may pay for itself in improving retention and lessening the need for expatriate recruitment.

As for the courses themselves, those who actually design and deliver these new programs will generally want them to be rigorous and equivalent to those in developed countries. There can be a tendency to add in extra “burdens” which seem a good idea at the time, but may be counterproductive if many trainees find themselves unable to complete their studies. When programs are developed, it should be taken into account “what trainees can bear”, especially given that those who do not complete training may be much more likely to leave the public system. Educators on the ground need to be fully aware of the clinical workloads of their students, their family and community commitments, and their financial struggles. Maintaining rigor while avoiding counterproductive overburdening is a difficult balance, especially at times when health systems or individual specialist departments are in crisis and when clinical workloads are especially high. Seeking input into course development from specialist advisors who have trained or worked in developed countries and are fully familiar with the rigor required, but who have also worked in specialist and other clinical roles in developing countries, may be of particular value. Recently, increasing numbers of developed country specialists and trainees are seeking out and training for roles in global health, and this may lead to the availability of more advisors who are able to come up with better solutions for addressing difficult issues such as the universal dilemma of rigor versus bearability in specialist training.

Along this line, planners should also give some thought to whether or not it is desirable for most enrollees to complete Master’s qualifications, and it would be useful for this to be explicitly understood from the start. This may vary from country to country. A “survival of the fittest” situation, where only a minority of enrollees attain full specialist qualifications, can be counterproductive in health systems where there are few options for those who do not fully complete training. While most Pacific Island countries have been able to incorporate Diploma graduates into specialist roles, the situation is quite different in Fiji, where the health system has fewer career pathways for those who have left training without a Master’s.

## Conclusion

The establishment of postgraduate specialist training in Fiji has had positive impact on health services in the Pacific (see Table 4). These graduates have had a highly positive impact on the staffing situation of specialist departments at the major hospitals in Fiji, and have greatly lessened the dependence on expatriate staff in Fiji and throughout the Pacific (see Table 4). Four Master’s graduates are now in senior academic positions at the Fiji School of Medicine, which has positive implications for the continuity of the training programs.

**Table 4:** Number of trainees who were awarded specialist Postgraduate Diplomas and Master’s in Medicine at the Fiji School of Medicine by specialty

**Specialty**	**Fiji**			**Other Pacific Islands**		
	**Postgraduate Diplomas**	**Current Master’s students**^**a**^	**Master**’**s in Medicine**	**Postgraduate Diplomas**	**Current Master’s students**^**a**^	**Master’s in Medicine**
Anesthesia	20	5	4	25	6	4
Internal Medicine	18	3	10	17	3	7
Obstetrics and Gynecology	15^b^	4	4	13^b^	4	7
Pediatrics/Child Health	25^b^	7	3	12	0	0
Surgery	26	11	4	23	5	7
Total	104^b^	30	25	90^b^	18	25

^a^Including current Master’s students, deferred students (one from another Pacific island), and students awaiting Master’s enrollment following the awarding of a Postgraduate Diploma (four from Fiji, two from other Pacific islands).

^b^Additionally, six Fiji trainees and four trainees from other Pacific islands who subsequently enrolled in the OBGYN Master’s received their Postgraduate Diplomas elsewhere, and three Fiji trainees who subsequently enrolled in the Pediatrics Master’s received their Diplomas elsewhere.

The establishment of these programs has not been without significant challenges and threats to their viability. Educational programs evolve over time and are impacted by national and international events, as well as by events within health systems themselves. While the earliest cohorts of trainees received their Master’s shortly after a particularly unstable time in Fiji, it is not uncommon for developing countries to undergo periods of relative instability. It is possible that careful attention to the issues raised above could have attenuated the dropouts and resignations that occurred for a number of years after the 2000 *coup d*’*etat*. Additionally, embedding the collection and analysis of enrollment and follow-up data into the design of training programs that are established can lead to early detection of difficulties. Qualitative data can provide additional insights. In particular, being aware of and addressing issues that are causing dissatisfaction can potentially lead to changes that improve retention, and perhaps prevent or attenuate downward spirals of resignations in troubled times.

In sharing our experiences, we would like to encourage others to add to global knowledge about how best to scale up specialist training in developing countries, and in particular to note whether and to what extent our findings have relevance in other countries and regions.

## Competing interests

The funders of the project had no role in study design, or collection, analysis or interpretation of data, or writing of the report, or the decision to submit the article for publication.

All researchers were independent from funders. KO and KU were employees of James Cook University and RM and ER were employees of the Fiji School of Medicine at the time the study was carried out. This did not impact on the study design, or collection, analysis or interpretation of data, or writing of the report, or the decision to submit the article for publication.

## Authors’ contributions

KO is the first author and guarantor. She completed the PhD study on which this manuscript is based. She designed the study, collected, entered and analyzed the qualitative and quantitative data, wrote drafts of the paper for circulation, and revised the manuscript based on the comments of co-authors and reviewers. KU was the PhD supervisor for the study from which this manuscript was drawn. She played a major role in study design and in the analysis and interpretation of data, as well as in commenting on the manuscript and giving final approval of the version that is being submitted. RM was based in Fiji during the time that the qualitative interviews were carried out. He provided input into study design, feedback on the interpretation of the data from a local perspective, as well as into revisions of the manuscript, and giving final approval of the version that is being submitted. ER has been based in Fiji continuously during the 12 years that the manuscript describes. She played a role in developing relevant areas of inquiry for the semi-structured interviews, as well as feedback on data interpretation from a local standpoint. She has also provided feedback and suggestions for revision of the manuscript, giving final approval of the version that is being submitted.

## Authors’ information

Kimberly Oman worked as an external advisor in Medicine at the Fiji School of Medicine from 1999 until 2001 and played an important role in the development of postgraduate training in Internal medicine. She is an honorary associate of the Fiji School of Medicine.

Kim Usher, Professor of Nursing at James Cook University in Townsville, Australia, has played a major role in the expansion and reform of nursing education in Fiji.

Robert Moulds worked as an external advisor in Medicine at the Fiji School of Medicine in 1999 and played a major role in the development of specialist training in Internal Medicine. He was employed as Professor of Medicine from 2002 until 2010 at the Fiji School of Medicine. He is currently a senior advisor at Therapeutic Guidelines in Melbourne, Australia.

Elizabeth Rodgers, Associate Professor of Pediatrics, is in charge of postgraduate specialist training at the Fiji School of Medicine, is head of the Pediatrics Department, and works as a pediatrician at Colonial War Memorial Hospital in Suva, Fiji.
